# Programmed Editing of Rice (*Oryza sativa* L.) *OsSPL16* Gene Using CRISPR/Cas9 Improves Grain Yield by Modulating the Expression of Pyruvate Enzymes and Cell Cycle Proteins

**DOI:** 10.3390/ijms22010249

**Published:** 2020-12-29

**Authors:** Babar Usman, Gul Nawaz, Neng Zhao, Shanyue Liao, Baoxiang Qin, Fang Liu, Yaoguang Liu, Rongbai Li

**Affiliations:** 1 State Key Laboratory for Conservation and Utilization of Subtropical Agro-Bioresources, College of Agriculture, Guangxi University, Nanning 530004, China; babarusman119@gmail.com (B.U.); gulnawazmalik@yahoo.com (G.N.); nengzhao@st.gxu.edu.cn (N.Z.); 1817303014@st.gxu.edu.cn (S.L.); bxqin@gxu.edu.cn (B.Q.); fangliu@gxu.edu.cn (F.L.); 2 State Key Laboratory for Conservation and Utilization of Subtropical Agricultural Bioresources, South China Agricultural University, Guangzhou 510642, China

**Keywords:** rice, CRISPR/Cas9, mutant, homozygous, heterozygous, grain size, proteins

## Abstract

Rice (*Oryza sativa* L.) is one of the major crops in the world and significant increase in grain yield is constant demand for breeders to meet the needs of a rapidly growing population. The size of grains is one of major components determining rice yield and a vital trait for domestication and breeding. To increase the grain size in rice, *OsSPL16/qGW8* was mutagenized through CRISPR/Cas9, and proteomic analysis was performed to reveal variations triggered by mutations. More specifically, mutants were generated with two separate guide RNAs targeting recognition sites on opposite strands and genomic insertions and deletions were characterized. Mutations followed Mendelian inheritance and homozygous and heterozygous mutants lacking any T-DNA and off-target effects were screened. The mutant lines showed a significant increase in grain yield without any change in other agronomic traits in T_0_, T_1_, and T_2_ generations. Proteomic screening found a total of 44 differentially expressed proteins (DEPs), out of which 33 and 11 were up and downregulated, respectively. Most of the DEPs related to pyruvate kinase, pyruvate dehydrogenase, and cell division and proliferation were upregulated in the mutant plants. Pathway analysis revealed that DEPs were enriched in the biosynthesis of secondary metabolites, pyruvate metabolism, glycolysis/gluconeogenesis, carbon metabolism, ubiquinone and other terpenoid-quinone biosynthesis, and citrate cycle. Gene Ontology (GO) analysis presented that most of the DEPs were involved in the pyruvate metabolic process and pyruvate dehydrogenase complex. Proteins related to pyruvate dehydrogenase E1 component subunit alpha-1 displayed higher interaction in the protein-protein interaction (PPI) network. Thus, the overall results revealed that CRISPR/Cas9-guided *OsSPL16* mutations have the potential to boost the grain yield of rice. Additionally, global proteome analysis has broad applications for discovering molecular components and dynamic regulation underlying the targeted gene mutations.

## 1. Introduction

Rice is one of the important food crops worldwide, providing the staple food for half of the global population. In recent years, with the rapid population growth, climate change, and increasing soil pollution, the effective planting area of crops has been decreasing year by year. Therefore, increasing rice production has become an important aspect to ensure food security. Rice yield is mainly determined by the effective panicle numbers (PN) per plant, grain number per panicle (GNPP), and the thousand-grain weight (GWT). Among them, the grain weight (GW) depends on the changes in grain length (GL), grain width (GWD), and grain thickness. Therefore, reasonable optimization of grain shape can provide an important guarantee for the cultivation of high-yielding varieties [1]. The genetic regulation pathways of rice grain size are complex and diverse, which may involve ubiquitinated protein degradation, plant hormones, and the interaction between different signaling pathways.

Many important agricultural trait-related genes provide valuable resources for the application of bioengineering methods to improve the grain yield of crops. In rice, various genes regulating grain shape and size have been functionally isolated. Among those genes, *GL3.1*, *GS3*, *TGW3*, and *TGW6* negatively regulates GL, whereas *OsLG3*, *OsLG3b*, and *GL4* positively regulate GL [2,3,4,5,6,7,8,9,10,11]. *GW2*, *TGW2*, and *GW5* negatively regulate GW, whereas *GW6a*, *GS5*, and *GW8* positively control GW [12,13,14,15,16,17,18]. *GSA1*, *GL2*, *GL6*, and *GLW7* positively regulates GL and GW [19,20,21,22,23]. *GS9* positively regulates GW but negatively regulates GL, whereas *GW7* negatively regulates GW but positively regulates GL [24,25,26]. Because of their effects on rice yield, these genes should be given more attention for their application in coping with food shortage. Although the natural mutations in these alleles have contributed to yield increase, recently potential application values in the improvement of grain size and yield in rice can be found by using the latest genome editing techniques.

The first cloned grain *GS3* encodes a transmembrane protein composed of four domains and its base mutations result in reduced grain size [2]. *GL3.1* encodes a Ser/Thr phosphatase and its single nucleotide polymorphism leads to changes in GL [3]. A large fragment DNA tandem repeats that occurred at the *GL7* locus determines long-grain phenotype. This genomic structural variation leads to an increase in gene expression and an increase in GL [25]. In previous work, it was identified that *GS2* encodes rice growth regulator *OsGRF4*, and rare dominant mutations in the miR396c target caused an increase in gene expression, which promotes cell division and growth and showed increased GL. *GW2* encodes cytoplasmic E3 ubiquitin ligase, which may be involved in the degradation of proteins that promote cell division and regulate grain size [12,27]. Thus, developing mutants of these genes may accelerate plant functional genomics research and amenability to efficient genetic modification.

The clustered regularly interspaced short palindromic repeats/CRISPR-associated protein 9 (CRISPR/Cas9) has appeared as a powerful tool in agriculture by helping to develop novel varieties with improved agronomic traits and revolutionized breeding system. CRISPR/Cas9 technology provides the fastest way to generate mutants having desired traits by introducing gain-of-function mutations or deleting negative elements controlling undesired traits through precise genome editing. The CRISPR/Cas9 system is an accurate, convenient, and high-efficiency biological genome editing method developed in recent years. This system only needs single guided RNA (sgRNA) and Cas9 nuclease to generate target gene site-directed mutations [28,29,30]. CRISPR/Cas9 is an adaptive immune system formed by bacteria and archaea to defend against foreign nucleic acid invasion. Because of its specificity, simplicity, and versatility, CRISPR/Cas9 is regarded as a new type of site-specific genome editing tool that is used for crop improvement and biological studies [31,32,33]. CRISPR/Cas9 can generate double-stranded breaks (DSB) in the target DNA which then repaired through non-homologous end joining (NHEJ) or homology-directed recombination (HDR) pathways resulting in insertion/deletion (INDELS) and substitution mutations [34]. For the design of the target site, a protospacer adjacent motif (PAM) sequence is required, which means that almost all genes in the genome can be targeted for editing by the CRISPR/Cas9 system. The CRISPR/Cas9 system can accurately and completely edit the target gene, and the mutations obtained are heritable [35]. Currently, the CRISPR/Cas9 system has been successfully applied to different plant species, including wheat [36], tobacco [37,38], Arabidopsis [37,39], corn [40,41], soybean [42], and rice [43,44,45,46,47,48,49,50]. For instance, Usman et al. [48] simultaneously mutagenized four genes in rice by utilizing the CRISPR/Cas9 technique, leading to increases in grain yield and fragrance. *GW5* is a positive regulator of brassinolide signaling and its CRISPR/Cas9 knockout mutants resulted in enhanced grain yield [51]. *GS5* regulates grain size by up-regulating the expression of cell cycle genes and promoting cell division [15]. CRISPR/Cas9-mediated editing of *OsPYL9*, *GS3*, *GW2*, and *GW5* has also given rise to yields in rice [17,50,52,53,54]. Rice grain yield has been improved by knocking out genes including *DEP1*, *Gn1a*, and *TGW6* that are known to be negative regulators of grain yield [55]. Four genes *Gn1a*, *DEP1*, *GS3*, and *IPA1* have been independently edited using the CRISPR/Cas9 system resulting in expected phenotypes such as enhanced grain number, dense erect panicles, and larger grain size [56]. In another study, the simultaneous mutation of *GW2*, *GW5*, and *TGW6* using CRISPR/Cas9 resulted in a 29.3% increase in GWT in the triple null mutant [57]. This suggests that pyramiding null mutants of major yield-related genes in a single cultivar via CRISPR/Cas9 would be crucial in regulating the yield components of rice. Therefore, the extensive usage of this system provides new scientific guidance for adopting advanced techniques to develop elite rice varieties. *OsSPL16* is synonymous with *GW8* which encodes a promoter binding protein that promotes cell division and increases GWD. Loss-of-function mutations of *OsSPL16* confer slender grain type and better quality of appearance in Basmati rice [16]. There are very few reports on the targeted editing of key genes that are important in rice breeding, and the breeding value of related mutants is lacking.

To investigate the myriad of mutations that affect biological function at whole proteome-wide, a high throughput technique is required which should enable the analysis of generated mutants. With the advances in molecular biology techniques, iTRAQ (isobaric tags for relative and absolute quantification)-based proteomic analysis may offer deep understandings about the potential molecular mechanism of mutations [58,59,60]. In rice, proteomic analysis has been successfully performed to screen the impact of mutations comprehensively [48,50]. Very recently, Usman et al. [48] conducted a proteome-wide analysis in both wild type (WT) and CRISPR/Cas9 rice mutants. In addition, Nawaz et al. [49] applied iTRAQ-based proteomics study in CRISPR/Cas9 rice mutants, including blast susceptible and highly resistant ones. These studies suggest that proteome analysis provides the complete depiction of structural and functional information of the response mechanism to mutations. Furthermore, the combination of proteomic with genome editing technologies has achieved success to screen the differential response of a large number of proteins in various crop plants.

In this study, the conventional Indica rice variety (VP4892) widely transplanted in Guangxi province, China, was used for the editing of *OsSPL16* and obtained a new set of mutant germplasm with increased yield and important breeding value. We designed target-specific primers and confirmed the mutation sequence by amplifying target regions of the gene. We successfully obtained homozygous and heterozygous mutants with enhanced grain yield. To gain more insights, we then performed iTRAQ-based proteomic analysis of a homozygous mutant line and analyzed the perturbations caused by mutations at proteome-wide and screened differentially expressed proteins (DEPs). In summary, we provided an example of how to improve the grain yield of rice through the use of the CRISPR/Cas9 system, which may offer new promises for rice breeding. The study of mutants will have access to further understand the regulation mechanism of rice grain development and provide a theoretical basis for traditional rice breeding.

## 2. Results

### 2.1. Validation of Targets Assembly and Genotyping of Mutant Plants

**Figure 1:**
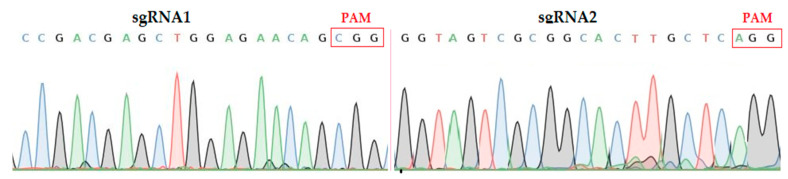
Sequencing chromatograms of both target sites assembled in vector. sgRNA; single guided RNA, PAM; protospacer adjacent motif.

Overlapping PCR was used to amplify the sgRNA expression cassette containing both targets. The amplified product was mixed and purified by TaKaRa MiniBEST Purification Kit Ver.4.0. The purified product was sequenced using specific primers (SPL1/SPR; Table S1). The results showed that the two target sequences assembled in the intermediate vector through the *Bsa* I site were consistent with the designed sgRNA sequences, so the constructed vector is suitable for the next step of Agrobacterium Mediated genetic transformation of rice (Figure 1).

### 2.2. Genotyping, and Protein Modeling

**Figure 2:**
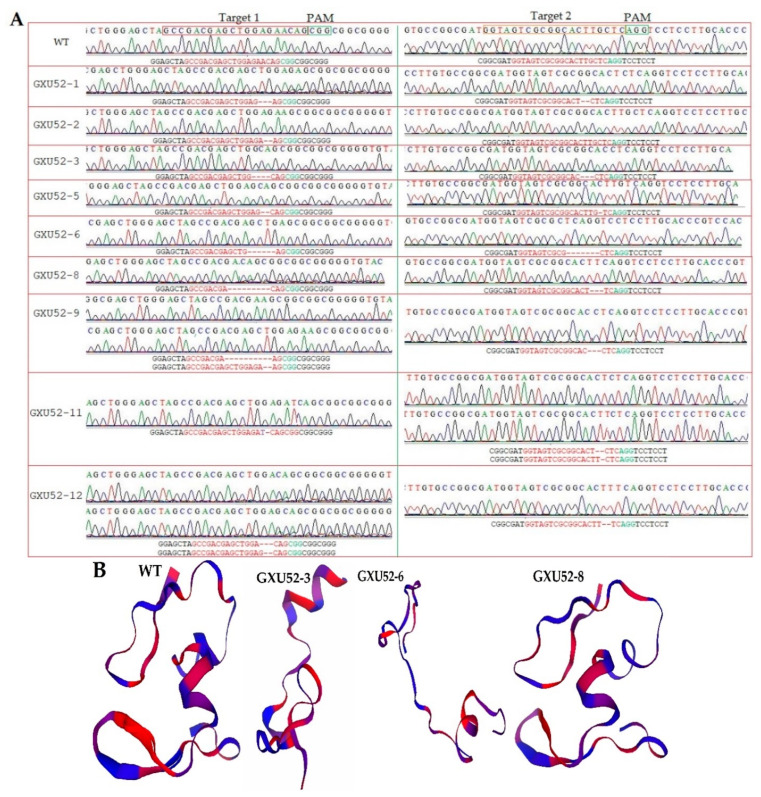
Sequence chromatograms and protein modeling of T_0_ mutant plants. (**A**) Sequencing peaks for wild type (WT) and mutant plants. Base deletion and insertion are represented with “-” and purple letters, respectively. Red and green letters are the target regions and protospacer adjacent motif (PAM) sequence, respectively; (**B**) the three-dimensional protein structures of the WT and mutant lines.

In total, 50 calli were treated with transformed *A. tumefaciens* and 12 rice plantlets were obtained. The DNA from each plantlet was extracted, and mutations were verified using target-specific primers (qGW8F/R; Table S1). The sequencing results displayed that 9 independent mutant lines showed mutations in target sites, representing an editing efficiency of 75%. The mutation patterns of T_0_ mutant lines resulted in homozygous, bi-allelic, and mono-allelic heterozygous plantlets. There were 4 mono-allelic heterozygous, 2 bi-allelic heterozygous, 3 homozygous, and 3 WT plantlets at the first target and 3 mono-allelic heterozygous, 1 bi-allelic heterozygous, 4 homozygous, and 4 WT plantlets at the second target. We found that two mutant plantlets (GXU52-6 and GXU52-8) presented homozygous mutations for the first and second target sites (Table S2). GXU52-6 displayed 6 bp and 7 bp deletions at the 1st and 2nd target positions, respectively. GXU52-8 presented 9 bp and 3 bp deletions at the 1st and 2nd target sites, respectively (Figure 2A).

We selected GXU52-3 (mono-allelic heterozygous), GXU52-6 (homozygous), and GXU52-8 (homozygous) lines for three-dimensional protein modeling. The three-dimensional protein structure of WT and mutant lines showed a significant difference (Figure 2B). Using the site-specific primers (POT1F/R-POT10F/R; Table S3), the DNA of 18 T_1_ mutant plants was amplified to evaluate the five most potential positions having higher ranking off-target potential. The predicted off-target regions were successfully amplified and there were no off-target mutations found in selected five loci against both targets (Table S4).

### 2.3. Screening and Segregation Analysis of Transgene-Free Plants

The T_1_ generation was evaluated to screen the transgene (T-DNA) free mutant plants. The cas9-specific (cas9-F/Cas9-R; Table S1) primers were used and a total of 18 mutant plants were used to detect the presence or absence of the exogenous DNA. We found that 8 plants were amplified to Cas9 vector sequence, whereas 10 plants were T-DNA-free (Figure S1). The segregation pattern of the progeny of the T_0_ generation was evaluated. Results revealed that homozygous mutations showed a similar mutation pattern in next-generation, whereas bi-allelic and mono-allelic heterozygous mutant lines followed the classic Mendelian inheritance pattern (1:2:1) (Table S5).

### 2.4. Agronomic Performance

**Figure 3:**
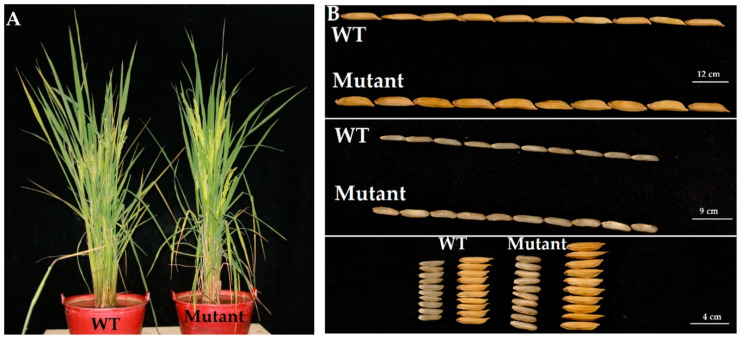
The plant **(A)** and grain phenotype **(B)** of wild type (WT) and mutant lines. Seeds were randomly collected from GXU52-3, GXU52-6, and GXU52-8 mutant lines for phenotyping.

We selected GXU52-3, GXU52-6, and GXU52-8 mutant lines for the investigations of agronomic traits. The results for T_0_, T_1_, and T_2_ generations revealed that yielding traits showed a significant difference between WT and mutant lines. Mutant lines showed significantly increased GWT, GWD, and yield per plant (YPP), whereas there was not any change in other agronomic traits like plant height (PH), PN, panicle length (PL), flag leaf length (FLL), flag leaf width (FLW), and GNPP (Table 1; Figure 3A). The GWT and GWD were increased from 29.8 g to 44.9 g, and 2.8 mm to 3.9 mm, respectively; the YPP was increased from 27.5 g to 42.9 g (Figure 3B). The T_1_ and T_2_ plants showed results were consistent with the T_0_ generation, which clearly showed that mutations showed an effect in subsequent generations (Table 1).

### 2.5. Peptide/Protein Identification and Screening of Differentially Expressed Proteins (DEPs)

**Figure 4:**
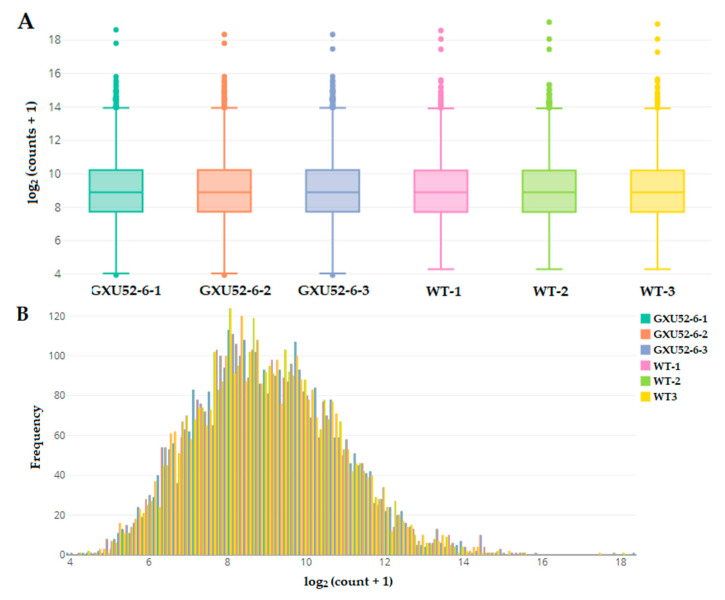
Basic information about proteomic analysis of the wild type (WT) and mutant line (GXU52-6). (**A**) Box-and-whisker plot of proteomic count data distribution with log2 values of the normalized abundance; (**B**) Histogram for count data distribution frequency of transformed data from WT and mutant line.

In total, 574,173 spectra were produced from the iTRAQ experiment. We identified 66,313 matched spectra, 26,986 peptides, 4752 total proteins, and 4743 protein after sorting deleting proteins with missing values. The count data distribution by box and whisker plot showed a clear difference between WT and GXU52-6 (Figure 4A). The histogram of protein count data presented that the protein frequency was higher at 6 to 12 of log_2_ (counts + 1) (Figure 4B). The information about identified peptides and proteins is given in Supplementary file 2.

Comparison analysis between WT vs GXU52-6 showed 44 DEPs, including 11 down-regulated and 33 up-regulated DEPs (Supplementary file 2). The DEPs were screened with a fold change (FC) of ≥1.20 (Student’s *t*-test, *p* ≤ 0.05). We found that DEPs related to pyruvate kinase (Q6AVA8, A0A0E0HXE6, Q259K4, A0A0E0IYE1, B8BM17, A0A0E0IZN3, Q8GVP6, and A0A0E0FK16), pyruvate dehydrogenase E1 (Q6Z5N4, A2Z2Z0, A2XPT6, B8B945, Q10G39, and B8BN11), dihydrolipoamide acetyltransferase component of pyruvate dehydrogenase complex (A0A0E0IJD8, Q2QWU7, A0A0E0IC70, and A0A0E0FKX9), acetyltransferase component of pyruvate dehydrogenase complex (A0A0E0FYX8, B8AGW7, and Q5VS74), mitochondrial pyruvate carrier (A0A0E0HZY5 and B8BEY3), 4-hydroxyphenylpyruvate dioxygenase (Q6H4V1), and dihydrolipoamide acetyltransferase component of pyruvate dehydrogenase complex (B9FTG2) were up-regulated. The DEPs Q7XE16, A2XAR1, and Q10CF3 related to the cell division cycle protein 48, proliferating cell nuclear antigen, and cell differentiation protein rcd1, respectively, were also up-regulated, whereas B3VMC0 (Betaine aldehyde dehydrogenase 2 (BADH 2) was down-regulated (Supplementary file 2).

### 2.6. Functional Assignment of the Differentially Expressed Proteins (DEPs)

**Figure 5:**
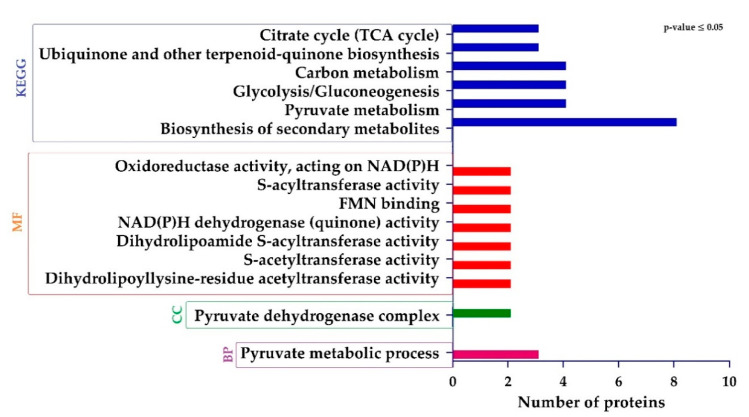
Histogram showing significantly enriched gene ontology (GO) annotations and Encyclopedia of Genes and Genomes (KEGG) pathways of differentially expressed proteins (DEPs).

Gene ontology (GO) and KEGG (Kyoto Encyclopedia of Genes and Genomes) analysis and was performed to assess the different functions of DEPs. The GO terms regarding “biological process (BP)”, showed that DEPs related to pyruvate metabolic process were significantly regulated. Regarding “cellular component (CC)”, proteins were only associated with pyruvate dehydrogenase complex were enriched. Finally, from the “molecular function (MF)” perspective, proteins involved in dihydrolipoyllysine-residue acetyltransferase activity, s-acetyltransferase activity, dihydrolipoamide s-acyltransferase activity, NAD(P)H dehydrogenase (quinone) activity, FMN binding, s-acyltransferase activity, and oxidoreductase activity, acting on NAD(P)H were sufficiently regulated. The KEGG pathway analysis showed that DEPs were involved in the biosynthesis of secondary metabolites, pyruvate metabolism, glycolysis/gluconeogenesis, carbon metabolism, ubiquinone and other terpenoid-quinone biosynthesis, and citrate cycle (TCA cycle) (Figure 5).

### 2.7. Functional Interaction Networks of the Differentially Expressed Proteins

**Figure 6:**
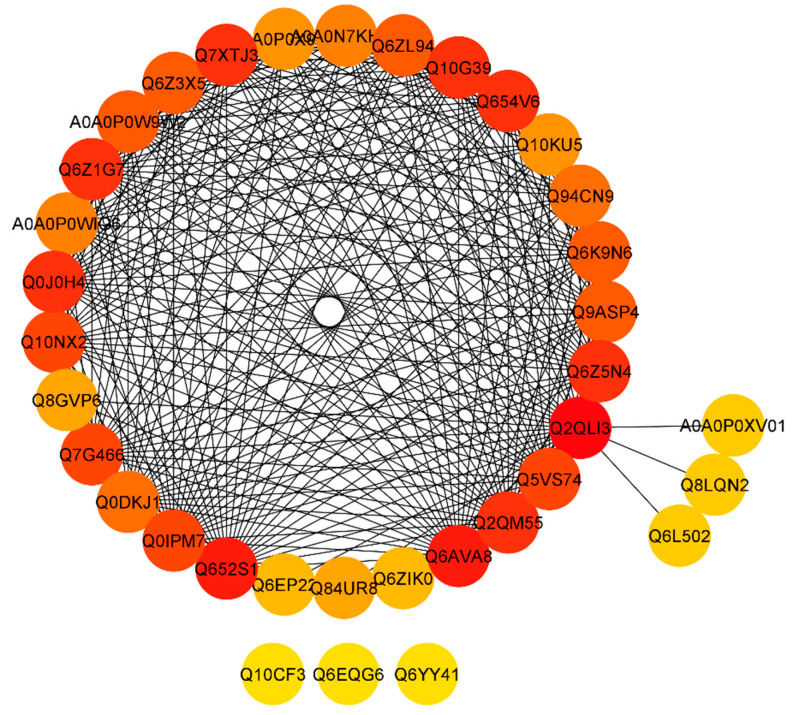
STRING software predicted protein to the protein network of differentially expressed proteins (DEPs). Differentially accumulated proteins are represented by a node, whereas the different color of lines represents evidence for the predicted functional relationship. The strong interaction is indicated by redder color. The proteins outside the circle showed no or weak interaction.

To retrieve a functional network of protein interactions, a Search Tool for the Retrieval of Interacting Genes/Proteins (STRING) database was used. After extracting the predicted network, we found that proteins including Os12g0641300 protein (Q2QLI3), Os06g0258900 protein; putative fructose/tagatose bisphosphate aldolase (Q652S1), and pyruvate dehydrogenase E1 component subunit alpha-1 proteins (Q6AVA8, Q6Z5N4, Q654V6, Q2QM55, Q0J0H4, Q6Z1G7, Q7XTJ3, and Q10G39) with a degree greater than 23 (Supplementary file 2). Some proteins including Putative cold shock protein-1 (Q84UR8), probable pyruvate, phosphate dikinase regulatory protein, chloroplastic (Q8GVP6), cell division inhibitor-like (Q6EP22), probable tocopherol O-methyltransferase (Q6ZIK0), Os10g0436800 protein (A0A0P0XV01), putative quinone-oxidoreductase (Q8LQN2), and putative GTP-binding protein (Q6L502) showed degree value of less than 5, whereas, cell differentiation protein rcd1 (Q10CF3), Os09g0345500 protein (Q6EQG6), and Putative UDP-glucosyltransferase (Q6YY41) showed 0 degree value and no interaction with other proteins (Figure 6; Supplementary file 2). The above results revealed that pyruvate dehydrogenase E1 component subunit alpha-1 proteins were found to highly interact.

### 2.8. RT-qPCR Based Analysis of OsSPL16 Expression and Proteomic Data Validation

**Figure 7:**
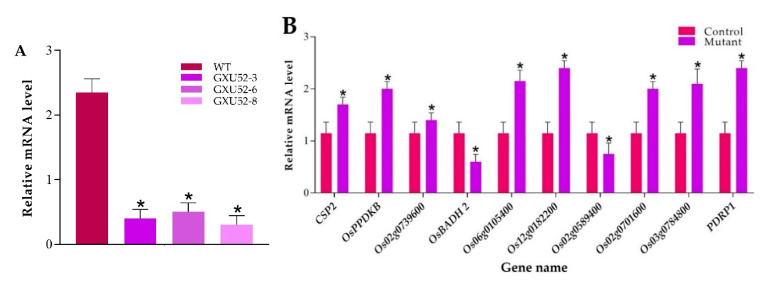
Real-time quantitative PCR validation of the *OsSPL16* expression level in wild type (WT) and mutant lines, and ten selected differentially expressed proteins (DEPs) responsive genes under normal conditions. (**A**) Expression analysis of *OsSPL16* in WT and mutant lines (**B**) Relative expression level of ten selected DEPs responsive genes. The data were analyzed by three independent repeats, and standard deviations were shown with error bars. Significant differences in expression level were indicated by “*”, Student’s *t*-test, *p ≤* 0.01.

The internal reference gene *Actin was* used for the normalization of expression between samples. The results exhibited that the *GW8* expression was significantly reduced in mutant lines as compared to WT (Figure 7A). To validate the results of the proteomic experiment in total, ten random genes were randomly selected encoding two downregulated proteins including betaine aldehyde dehydrogenase 2 (*OsBADH2*) and glycosyltransferase (*Os02g0589400*), and eight up-regulated proteins including, probable pyruvate, phosphate dikinase regulatory protein (*PDRP1*), cold shock domain protein 2 (*CSP2*), pyruvate, phosphate dikinase 1 (*OsPPDKB*), pyruvate dehydrogenase E1 component subunit alpha-1 (*Os02g0739600*), acetyltransferase component of pyruvate dehydrogenase complex (*Os06g0105400*), dihydrolipoamide acetyltransferase component of pyruvate dehydrogenase complex (*Os12g0182200*), probable tocopherol O-methyltransferase, chloroplastic (*Os02g0701600*), and cell differentiation protein rcd1 (*Os03g0784800*). The RT-qPCR results represent that the expression pattern of selected genes was correlated with the mass-spectrometry data (Figure 7B).

## 3. Discussion

Rice is not only an important food crop but also a model plant for functional genomics. High yield has always been an important goal of rice breeding, and the grain size is one of the imperative factors affecting rice yield. Therefore, an in-depth study of the molecular mechanism of rice grain type changes will not only help to further understand the mechanism of rice yield improvement but also provide new genetic resources for rice breeding, which is of great significance. The selection and application of high yielding rice varieties is a breakthrough in the field of breeding. The rise of modern molecular biotechnology provides some quicker and more efficient methods for rice breeding. CRISPR/Cas9 is an evolving gene-directed editing technology with high specificity and editing efficiency. Compared with zinc-finger nucleases (ZFNs) [61,62,63] and TALE nucleases (transcription activator-like effector nucleases, TALENs) [64,65,66], CRISPR/Cas9 is more powerful, simple, and flexible.

The basis for the cultivation of new rice varieties lies in the existing high-quality germplasm resources, and the key to its success lies in the accurate selection of suitable genes for modification. There are both positive and negative regulatory genes in plant growth and development, resistance to adverse environments, and grain yield. Therefore, it is possible to consider introducing mutations in positive regulatory genes or knocking out negative regulatory genes to improve rice grain yield. According to the current level of technology, the gene knock-out efficiency of CRISPR/Cas9 in rice is much higher than that of gene knock-in/replacement based on this technology [48,49,50]. Therefore, selecting rice specific regulatory genes for targeted mutagenesis is the most concise and effective strategy at present. Many yield-related genes have been mutagenized and their functions have been explored using CRISPR/Cas9 [55].

In this study, two sgRNAs were designed to perform targeted editing of the *OsSPL16* gene, and different types of mutants were obtained successfully in the T_0_ generation. The results of this study show that CRISPR/Cas9 can effectively edit rice targeted DNA sequences with high efficiency. Moreover, the same target site can produce multiple mutation types. Base deletion or insertion occurs near to PAM site, and the proportion of base deletions and type of mutation in T_0_ is unpredictable. The mutation frequency of *OsSPL16* in the T_0_ generation was as high as 75%, and homozygous mutations were achieved, indicating that multi-target targeted editing is beneficial to obtain homozygous mutations. The previous studies also revealed that the CRISPR/Cas9 generates the homozygous mutants in T_0_ generation and mutations mainly take place in transformed calli cells [48]. During genome editing experiments, CRISPR/Cas9 also requires consideration to avoid random mutations. According to the previous, there is a low possibility of off-target mutations in rice [48,49,50], but to satisfy the basic rules for biosafety, it is critical to assess and select the T-DNA-free mutants. We also followed the genetically modified (GM) rules to avoid public controversy and social acceptance issues. In this study, we screened the T-DNA-free lines and also verified the off-target free mutant plants by selecting the five most likely putative off-target sites against both targets. The segregation pattern of mutants and predictable inheritance in the subsequent generation is very important in basic research and molecular breeding. Previous studies observed that the Cas9-induced mutations were stable and inheritable to the next generations in a similar pattern as unmutated loci follow [50]. In this study, the PCR detection results of the target positions confirmed the inheritable mutations and segregation according to Mendel’s law. Previous studies found that using CRISPR/Cas9 technology, homozygous mutants can be obtained in the T_0_ generation, and these mutations can be stably passed on to the next generation. It is found previously that the mutation of the T_1_ generation is uncertain, and the mutation can be stably inherited after the T_2_ generation [67]. In addition, we also found that the position of the gene sequence targeted by the CRISPR/Cas9 gene-editing vector results in a different type of mutant plants. It can be seen from the above results that genetic materials developed based on CRISPR/Cas9 gene-editing technology provide abundant genetic materials for further research. It is worth noting that some of the materials obtained by CRISPR/Cas9 have clear genotype mutations, but there are no obvious phenotypic differences. This phenomenon reminds us that using CRISPR/Cas9 technology can not only develop basic materials for gene function research but also has great breeding application value.

In this study, we found that mutant plants showed increased GWD compared with the WT. These results confirmed that *OsSPL16* has the function on regulation GWD, and the effect of *OsSPL16* mutation on the advances in GWD was significant. GWT of *OsSPL16* mutants was significantly higher than those of WT plants. These results demonstrated that *OsSPL16* was successfully edited with the CRISPR/Cas9 system, and the mutants exhibited larger grain sizes. Thus, mutations of *OsSPL16* would be of great significance for grain yield improvement by increasing GW.

The size of the grain is one of the imperative characters for defining rice quality. Recently, many genes were characterized by pleiotropic effects on grain size and appearance. *GW2* loss of function mutants results in improved chalkiness percentage and GW [12]. *GW8* and *GS9* mutations via CRISPR/Cas9 contribute to and better appearance quality and formation of more slender grains in rice [16,26]. In this study, the GWD and GWT of the mutant were higher, suggesting that mutations of *OsSPL16* would be used for the improvement of grain appearance. Most of the grain size QTLs commonly exist with pleiotropic effects on other important agronomic traits. Mao et al. [2] reported that *GS3* regulates grain size its overexpression results in shortened leaf. Our study showed that mutation in *OsSPL16* led to enhanced grain yield without any change in FLL and FLW. Traditional breeding and marker-assisted strategy are time-consuming and laborious to breed lines with improved grain size. However, in this study, we verified that CRISPR/Cas9- mediated genome editing is an effective way to get the desired trait in one generation. CRISPR/Cas9 based editing of *OsSPL16* exhibited high editing efficiency in this knockout system due to easy resulting in a frameshift and premature termination. Further, marker-free knockout lines were screened in the T_1_ generation, which will provide more possibility to be used in rice breeding. Using CRISPR/Cas9, more favorable alleles with potential application values in the improvement of grain size and yield in rice can be achieved.

In this work, the comparative iTRAQ-proteomics analysis was performed to identify the changes proteome-wide. After screening the DEPs, we found that proteins related to pyruvate kinase and dehydrogenase, cell division, and differentiation were up-regulated in mutant plants. The KEGG and GO analysis also showed that proteins were enriched in pyruvate dehydrogenase complex and pyruvate metabolism. In PPI-network, the pyruvate dehydrogenase E1 component subunit alpha-1 proteins showed the highest interaction. The Betaine aldehyde dehydrogenase 2 (BADH 2) was down-regulated. Pyruvate kinase (PK) is a key enzyme that regulates the glycolysis pathway and synthesizes pyruvate and ATP (adenosine triphosphate) by catalyzing the irreversible transfer of the high-energy phosphate group from phosphoenolpyruvate (PEP) to ADP (adenosine diphosphate) [68,69]. Cotton and rice have 19 and 11 genes, respectively, which encode putative PK isozymes [70,71]. A previous study found that a novel rice white-core endosperm and defective grain filling mutant, *ospk2*, display significantly lower GW, starch content, and alteration of starch physicochemical properties when compared to WT grains [72]. We can attribute that functional cross-talk maybe exists between the cell cycle and pyruvate enzyme proteins. Thus, different functional changes generated by the mutations analyzed here could directly and/or indirectly contribute to the mutant’s phenotype. The regulation of these proteins is likely responsive to many other factors in addition to the cell cycle. Alternatively, the relatively increased level of expression of these proteins may be advantageous in cell proliferation.

In recent years, many studies have been done on the regulation of grain size. Some studies have pointed out that organ size is determined by the number of cells and cell size. Many genes have been cloned and studied, most of which regulate the grain size by controlling cell proliferation and expansion in spikelet hulls, such as *GW2*, *TGW2*, *GW5*, *GW6a*, *GS5*, and *GW8* [12,13,14,15,16,17,18]. Corresponding to this cell proliferation and cell elongation is the basic process for organ development. Proteins related to cell division, proliferation, and differentiation play a great role in rice grain development. Proliferating cell nuclear antigen is involved in DNA repair and cell cycle regulation [73]. Studies have found that *GS3*, *GL3.1*, *GL7*, *OsSPL13*, and *DEP1* have been reported to regulate GL via the regulation of cell division and expansion [2,3,21,25,74], whereas *GW2*, *qSW5*, *GW5*, *GS5*, *GW7*, and *GW8* regulate GWD through stimulation of cell division [12,14,15,16,24,51]. Other genes such as *OsBG1*, *OsMKK4*, and *OsMAPK6* promote cell division, and both GL and GWD [75,76,77]. *GS3*, a major QTL is a negative regulator of GL by regulating cell division [2]. The homolog of *GS3* in *Arabidopsis* is *AGG3*, which also control seed and organ size by regulating cell division [78]. *GL7* is a positive factor for GL which triggers and regulates cell expansion [25]. *GW2*, which, first being found as a major QTL for GWD, functions as a negative factor for GWD by mediating the degradation of the substrate in cell division [12]. In short, mutations in *OsSPL16* influence the expression of pyruvate kinase and cell cycle proteins. The expression levels of the cell cycle proteins in mutants were significantly increased compared with the WT, which was consistent with expectations. However, the upregulation of pyruvate kinase and cell cycle proteins together displayed that there may be a functional relationship between the expression of these proteins. Therefore, this study believes that mutant regulates rice grain yield by triggering the expression of pyruvate kinase and cell cycle proteins.

As a model plant, rice has the advantages of a simple genome, high seed setting rate, and simple genetic manipulation. The present work offers a unique genetic mechanism underlying rice yield improvement. Previously, some studies have been found on *OsSPL16* mutants, but no work exists which provides information about CRISPR/Cas9 mutants and proteome-wide assessment of mutation effects. The test results of mutants obtained from the successful editing of the rice *OsSPL16* gene in this study show that CRISPR/Cas9 can be successfully applied to the editing and modification of plant yield-related genes to achieve a better yield. At the same time, the application of proteomic technology can provide deep insights to reveal the changes at the whole proteome level. Additional work is necessary to explore the regulatory mechanism underlying the pyruvate kinase and cell cycle proteins to formulate approaches for enhancing the rice yield. Taking rice breeding for yield improvement as an example, there are many genes which are the negative regulator of grain yield. According to the results of existing research, targeted editing of these genes may help to improve rice yield. In addition, we can see from our research that for the same gene, the editing at the same site can also produce different types of mutations. Therefore, based on the existing germplasm to modify these target sites, it is obvious that it can help us to derive a series of new germplasm at relatively high efficiency, thereby speeding up rice breeding for yield improvement. This study shows that CRISPR/Cas9 based genome editing has important scientific significance in the research and application to reveal new insights into molecular regulation mechanisms.

## 4. Materials and Methods

### 4.1. Material Used and Experimental Conditions

The Indica rice variety VP4892 was selected for genetic transformation and seeds were collected from Rice Research Institute of Guangxi University. The CRISPR/Cas9 intermediate vector pYLCRISPR/Cas9pubiH and gRNA promoters (U6a and U6b) used in this study were provided by Professor Liu Yaoguang from South China Agricultural University, Guangzhou, China. The Oligo sequences and detection primers were synthesized by Beijing Genomics Institute, Beijing, China.

### 4.2. Target Site Selection and Vector Construction

**Figure 8:**
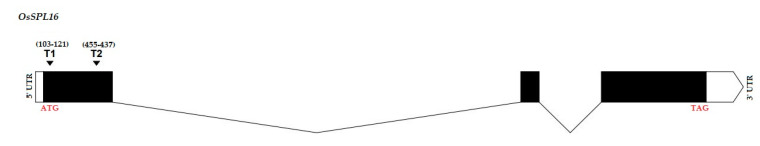
Location of both single guiding RNAs (sgRNAs) in *OsSPL16* gene. T1 and T2, represent first and second target sites, respectively. The black boxes represent codon regions.

According to the *OsSPL16* (*LOC_Os08g41940*) sequence provided by China Rice Data Center (http://www.ricedata.cn/gene/), the amplification of the *OsSPL16* gene was performed for VP4892 variety by using specific primers (GW8F/R). The *OsSPL16* gene is located on chromosome 8, has a total length of 5032 bp. This reference sequence was used to design oligonucleotide primers for the amplification of *OsSPL16* target positions. After confirming the gene sequence, two 20 bp long sgRNAs sequences followed by PAM (protospacer adjacent motif) were designed according to the A/G(N) 20NGG in the first exon region by using CRISPR-GE (http://skl.scau.edu.cn/) online tool [79] (Figure 8; Table S6). The designed sgRNAs sequences were blasted against the rice genome using NCBI (https://blast.ncbi.nlm.nih.gov/Blast.cgi) to exclude non-specific target sites and confirm specificity. The secondary structure of both sgRNAs was developed by RNAfold web server **(**http://rna.tbi.univie.ac.at/cgi-bin/RNAWebSuite/RNAfold.cgi) (Figure S2). We selected five potential off-targets containing at least two nucleotide mismatches for each target to analyze off-target effects (Table S3).

### 4.3. Construction of Vector and Rice Transformation

The expression cassette was generated according to Ma et al. [80], with slight modifications. The corresponding adapter primers Wx-U6-F/Wx-U6-R were used to construct the ligation reaction of the sgRNA expression cassette. Then, the ligation product was used as a template for PCR amplification. The product of the expression cassette was purified. A 15 μL restriction digestion ligation reaction was prepared, including pYLCRISPR/Cas9 plasmid 60 ng, 10 ng sgRNA-osU6a expression cassette mixture, 10 × CutSmart Buffer, 10 mM ATP, 10U BsaI-HF, 35UT4 DNA ligase, and variable temperature cycling for 15 cycles of digestion. The vector fragments were ligated with T4 DNA ligase (NEB) at 20 °C for 2 h. After the ligation product is transformed into DH5α competent cells by heat shock method, the product was cultured overnight. Colony PCR was performed, and the product was sent to BGI for sequencing. The ligated product was transformed by the heat shock method to competent cells of *E. coli* DH5α. The expression cassette was transformed into *A. tumefaciens* EHA105 by electroporation and rice transformation was achieved following Hiei et al. [81].

### 4.4. Genotyping, Phenotypic and Screening of T-DNA-Free Plants

The target sites of the T_0_, T_1,_ and T_2_ generations of the genetic transformation material were sequenced and analyzed. The sequencing files were processed using the DSDecode M tool [82] to view the deletions, insertions, substitutions, and type of mutations. The cetyltrimethylammonium bromide (CTAB) method was used to extract the genomic DNA from the fresh leaves of the mutant plants, and site-specific primers (GW-F/R) were designed to detect the on-target mutations. The agronomic traits such as PH, PN, PL, FLL, FLW, GNPP, GL, GWT, GWD, and YPP of WT and mutant lines were recorded in T_0_, T_1_, and T_2_ generations. The Cas9 gene-specific primers Cas9-F/Cas9-R were used to PCR-amplify the genomic DNA of mutant plants to screen T-DNA-free plants. PCR amplification conditions were as follows: initial denaturation at 95 °C for 5 min; followed by 36 cycles of denaturation at 95 °C for 30 s, annealing at 57 °C for 30 s, extension at 72 °C for 30 s; followed by a final extension at 72 °C for 5 min. The PCR products were detected by agarose gel electrophoresis. The plants that failed to amplify were considered as T-DNA-free.

### 4.5. Protein Preparation, Labeling, and Fractionation

Leaf samples (1 g) from WT and mutant plants were collected at the seedling stage at the same time point. The leaf samples were ground by liquid nitrogen and lysis, and protein extraction was performed according to Wang et al. (2014) [83]. The cells were suspended in lysis buffer (7 mol/L urea, 2 mol/L thiourea, 4% CHAPS, 40 mmol/L Tris-HCl, pH 8.5, 1 mM PMSF, 2 mmol/L EDTA) and sonicated in ice. A 100 μg of total protein was taken, and Trypsin Gold solution (sample:reagent = 30:1) was added and digested at 37 °C for 16 h. The sample was vacuum centrifuged and dried to obtain peptide dry powder. The peptide was reconstituted with 0.5 mol/L triethylamine borane (TEAB), and then labeled according to the iTRAQ Reagen t8-plex kit (SCIEX, Madison, WI, USA) following manufacturer instruction. For SCX chromatographic analysis, LC-20AB HPLC (Shimadzu, Kyoto, Japan) high-performance liquid chromatography system was used following the manufacturer’s instructions. The samples are marked with iTRAQ tags as follows: WT (113) and GXU52-6 (114).

### 4.6. Liquid Chromatography/Electrospray Ionization Tandem Mass Spectroscopy (LC-ESI-MS/MS) Analysis

The LC-20AD nano-high performance HPLC liquid system (Shimadzu, Kyoto, Japan) was used to suspend the peptide in buffer A according to the instructions, and the final concentration was about 0.5 μg/μL. 10 μL of supernatant with an automatic sampling system was aspirated and placed on a 2 cm C18 trapping column, and then eluted into a 10 cm C18 analytical column (inner diameter 75 μm). The samples were loaded at a rate of 8 μL/min for 4 min, then a concentration gradient of 2% to 35% of buffer B (98% ACN, 0.1% FA) was used to run at a flow rate of 300 nL/min for 44 min; then at 2 min the concentration of Buffer B was increased to 80% within 4 min, and finally, the concentration of Buffer B was reduced to 5% within 1 min. The peptides were separated by a nano-liquid chromatography system (HPLC), which was directly connected to the Q-EXACTIVE mass spectrometer (MS, ThermoFisher Scientific, San Jose, CA, USA), and the separated samples were analyzed by the Q-EXACTIVE mass spectrometer. For MS scanning, the m/z range of the primary MS full scan was 350–2000 u, and the m/z range of the secondary MS full scan was 100–1800 u.

### 4.7. Proteomic Data Analysis

Proteome Discoverer 1.2 (PD 1.2, Thermo) software was used to convert the raw mass spectrum data obtained from Orbitrap into MGF files. For protein identification, Mascot software (Version 2.3.02, Matrix Science Inc., Boston, MA, USA) was used for database search. For protein identification, the complete peptide matching error is controlled within 20 mg/L, the fragment matching error was controlled within 0.02 u, and trypsin digestion allowed up to 1 misalignment. To reduce the false positives of peptide identification, the protein to be identified must meet the confidence interval of Mascot probability analysis software to identify peptides of more than 99%, and the significance score within the confidence interval was ≥20. A 1.2-fold cutoff was set for quantitative changes to assess up-regulated and down-regulated proteins. The GO (http://geneontology.org/) and KEGG database (http://www.genome.jp/kegg/pathway.html) were used to clarify the biological and functional characteristics of DEPs. STRING database (version 10.0) (https://string-db.org/) was used for the PPI interaction network. Finally, Cytoscape 3.8.0 was used to visualize the PPI network.

### 4.8. *OsSPL16* Expression Analysis and Validation of Proteomic Data

The RNA prep pure Plant Kit (Tiangen Biotech. Co. Ltd., Beijing, China) was used to extract the total RNA from the leaves of WT and mutant lines. PrimeScript RT Reagent Kit with gDNA Eraser (TaKaRa, Kusatsu, Shiga, Japan) was used for reverse transcription to get the first strand of cDNA. The 10 μL RT-qPCR was set up, which includes 1 μL of cDNA template, 5 μL of 2×SYBR qPCR Mix (ToYoBo, Osaka, Japan), 0.5 μL of forward and reverse primers (10 μmol/L), and ddH_2_O to make up to 10 μL. The RT-qPCR amplification program was as follows: pre-denaturation at 95 °C for 1.5 min; 10 s at 95 °C, 30 s at 60 °C, and 20 s at 72 °C, a total of 40 cycles. The primers for RT-qPCR are listed in Table S7. To verify the results of iTRAQ, ten candidate genes were selected randomly. The relative expression level was calculated using the 2^−ΔΔ^*^C^*^t^ method [84].

### 4.9. Data Analysis

Agronomic data were analyzed with SPSS 20.0 software, using Student’s t-test and presented as the mean ± SD (*p* ≤ 0.05). The graphs for agronomic data and proteomic data were developed by GraphPad Prism (version 7.0, GraphPad Software Inc., San Diego, CA, USA).

## 5. Conclusions

In this experiment, the CRISPR/Cas9 system was effectively utilized to edit the *OsSPL16* gene individually and iTRAQ technology was also employed to analyze the proteome expression differences between WT and mutant plants. The characterization of noteworthy proteome-wide responses of mutants provides new understandings into rice yield improvement. The targeted multiplex genome editing also facilitated the identification of some candidate proteins and biological pathways that may involve in rice grain development. The upregulation of the pyruvate kinase and cell cycle proteins in *OsSPL16* mutants will provide a deep focus in the molecular breeding of rice. The mutant materials in this study can provide important germplasm resources for studying rice yield improvement, and at the same time provide a reference for the important DEPs. The identified candidate proteins and biological pathways might be directly or indirectly involved in rice yield improvement. The DEPs observed in this study can be further analyzed to reveal their functional role in rice. The targeted genome editing also facilitated a pathway-level study for engineered rice mutants with enhanced grain yield. CRISPR/Cas9 could be a promising approach to overcome the barriers in conventional breeding to improve grain yield. These results provide comprehensive insights into the molecular mechanism underlying the *OsSPL16* mutations. The *OsSPL16* mutants laid an imperative material foundation for additional application in stable and high yield breeding of rice. It provides a reference for the development of rice germplasm with important application value in production and is probable to provide an efficient way for germplasm resource innovation and practical significance.

## Figures and Tables

**Table 1:** Increased yield of mutant lines in T_0_, T_1_, and T_2_ generations.

Generation	Genotypes	PH	PN	PL	FLL	FLW	GNPP	GL	GWD	GWT	YPP
T_0_	WT	126.2 ± 4.4	8.5 ± 1.3	28.5 ± 1.4	56.4 ± 2.8	2.3 ± 0.2	143 ± 10	9.9 ± 0.1	2.9 ± 0.2	30.3 ± 1.3	27.5 ± 2.9
	GXU52-3	128.1 ± 3.7 ^ns^	8.6 ± 1.2 ^ns^	29.2 ± 1.8 ^ns^	55.9 ± 3.0 ^ns^	2.2 ± 0.4 ^ns^	143 ± 09 ^ns^	10.1 ± 0.3 ^ns^	3.9 ± 0.2 *	43.9 ± 1.4 *	41.7 ± 1.6 *
	GXU52-6	126.5 ± 4.5 ^ns^	8.7 ± 2.0 ^ns^	28.6 ± 1.6 ^ns^	57.0 ± 3.2 ^ns^	2.4 ± 0.3 ^ns^	145 ± 08 ^ns^	9.8 ± 0.4 ^ns^	3.8 ± 0.1 *	44.5 ± 1.2 *	42.9 ± 2.2 *
	GXU52-8	127.4 ± 3.7 ^ns^	8.8 ± 1.1 ^ns^	27.6 ± 1.5 ^ns^	56.8 ± 2.1 ^ns^	2.3 ± 0.4 ^ns^	143 ± 10 ^ns^	10.2 ± 0.3 ^ns^	3.9 ± 0.3 *	44.6 ± 1.5 *	42.5 ± 1.3 *
T_1_	WT	127.5 ± 2.5	8.7 ± 1.3	27.6 ± 1.3	55.9 ± 2.6	2.4 ± 0.2	145 ± 08	10.1 ± 0.3	2.9 ± 0.1	29.8 ± 1.4	28.2 ± 2.3
	GXU52-3-1	127.6 ± 4.3 ^ns^	8.5 ± 1.4 ^ns^	28.3 ± 1.5 ^ns^	56.8 ± 3.2 ^ns^	2.3 ± 0.3 ^ns^	144 ± 11 ^ns^	10.2 ± 0.2 ^ns^	3.8 ± 0.3 *	44.8 ± 1.5 *	42.1 ± 1.3 *
	GXU52-6-1	128.2 ± 5.5 ^ns^	8.8 ± 1.8 ^ns^	29.2 ± 1.3 ^ns^	57.1 ± 2.7 ^ns^	2.5 ± 0.2 ^ns^	143 ± 07 ^ns^	10.3 ± 0.2 ^ns^	3.9 ± 0.2 *	44.6 ± 1.3 *	41.9 ± 3.2 *
	GXU52-8-1	127.7 ± 2.2 ^ns^	8.4 ± 1.2 ^ns^	28.7 ± 1.4 ^ns^	57.2 ± 2.2 ^ns^	2.2 ± 0.1 ^ns^	144 ± 10 ^ns^	10.1 ± 0.4 ^ns^	3.9 ± 0.2 *	43.9 ± 1.2 *	42.3 ± 1.6 *
T_2_	WT	127.3 ± 4.7	8.6 ± 1.3	28.6 ± 1.2	57.2 ± 3.1	2.2 ± 0.3	144 ± 08	10.3 ± 0.1	2.8 ± 0.3	31.1 ± 1.1	28.9 ± 2.5
	GXU52-3-2	127.7 ± 5.4 ^ns^	8.7 ± 1.4 ^ns^	28.9 ± 1.5 ^ns^	56.7 ± 2.7 ^ns^	2.5 ± 0.4 ^ns^	143 ± 10 ^ns^	10.2 ± 0.2 ^ns^	3.9 ± 0.2 *	44.9 ± 1.3 *	41.9 ± 2.6 *
	GXU52-6-2	126.8 ± 4.5 ^ns^	8.5 ± 1.6 ^ns^	28.3 ± 1.4 ^ns^	57.3 ± 2.5 ^ns^	2.3 ± 0.4 ^ns^	145 ± 09 ^ns^	10.1 ± 0.3 ^ns^	3.8 ± 0.1 *	44.7 ± 1.6 *	42.8 ± 2.1 *
	GXU52-8-2	126.6 ± 3.7 ^ns^	8.4 ± 1.3 ^ns^	27.5 ± 1.7 ^ns^	56.7 ± 2.8 ^ns^	2.4 ± 0.3 ^ns^	146 ± 07 ^ns^	9.9 ± 0.2 ^ns^	3.7 ± 0.3 *	44.7 ± 1.4 *	42.4 ± 1.8 *

WT (wild type); PH (plant height) cm; PN (panicle numbers); PL (panicle length) cm; FLL (flag leaf length) cm; FLW (flag leaf width) cm; GNPP (grain number per panicle); GL (grain length) mm; GWD (grain width) mm; GWT (1000-grain weight) g; YPP (yield per plant) g. Five independent plants were used to collect data from three replicates (*n* = 5). * and ^ns^ denote the significant and non-significant differences (Student’s *t*-test, *p* < 0.01), respectively.

## Data Availability

Raw data can be provided to researchers interested on request to the corresponding author.

## Supplementary Materials

Supplementary materials can be found at https://www.mdpi.com/1422-0067/22/1/249/s1.

## Abbreviations

CRISPR	Clustered regularly interspaced short palindromic repeats
Cas9	CRISPR-associated protein 9
iTRAQ	Isobaric tags for relative and absolute quantitation
DEPs	Differentially expressed proteins
KEGG	Kyoto Encyclopedia of Genes and Genomes
GO	Gene Ontology

## References

[1] Xing Y., Zhang Q. (2010). Genetic and molecular bases of rice yield. Ann. Rev. Plant Biol..

[2] Mao H., Sun S., Yao J., Wang C., Yu S., Xu C., Li X., Zhang Q. (2010). Linking differential domain functions of the *GS3* protein to natural variation of grain size in rice. Proc. Natl. Acad. Sci. USA.

[3] Qi P., Lin Y.S., Song X.J., Shen J.B., Huang W., Shan J.X., Zhu M.Z., Jiang L., Gao J.P., Lin H.X. (2012). The novel quantitative trait locus *GL3. 1* controls rice grain size and yield by regulating Cyclin-T1; 3. Cell Res..

[4] Zhang X., Wang J., Huang J., Lan H., Wang C., Yin C., Wu Y., Tang H., Qian Q., Li J. (2012). Rare allele of *OsPPKL1* associated with grain length causes extra-large grain and a significant yield increase in rice. Proc. Natl. Acad. Sci. USA.

[5] Ishimaru K., Hirotsu N., Madoka Y., Murakami N., Hara N., Onodera H., Kashiwagi T., Ujiie K., Shimizu B.I., Onishi A. (2013). Loss of function of the IAA-glucose hydrolase gene *TGW6* enhances rice grain weight and increases yield. Nat. Genet..

[6] Wu W., Liu X., Wang M., Meyer R.S., Luo X., Ndjiondjop M.N., Tan L., Zhang J., Wu J., Cai H. (2017). A single-nucleotide polymorphism causes smaller grain size and loss of seed shattering during African rice domestication. Nat. Plants.

[7] Yu J., Xiong H., Zhu X., Zhang H., Li H., Miao J., Wang W., Tang Z., Zhang Z., Yao G. (2017). *OsLG3* contributing to rice grain length and yield was mined by Ho-LAMap. BMC Biol..

[8] Liu Q., Han R., Wu K., Zhang J., Ye Y., Wang S., Chen J., Pan Y., Li Q., Xu X. (2018). G-protein βγ subunits determine grain size through interaction with MADS-domain transcription factors in rice. Nat. Comm..

[9] Xia D., Zhou H., Liu R., Dan W., Li P., Wu B., Chen J., Wang L., Gao G., Zhang Q. (2018). *GL3. 3*, a novel QTL encoding a GSK3/SHAGGY-like kinase, epistatically interacts with *GS3* to produce extra-long grains in rice. Mol. Plant.

[10] Ying J.Z., Ma M., Bai C., Huang X.H., Liu J.L., Fan Y.Y., Song X.J. (2018). *TGW3*, a major QTL that negatively modulates grain length and weight in rice. Mol. Plant.

[11] Yu J., Miao J., Zhang Z., Xiong H., Zhu X., Sun X., Pan Y., Liang Y., Zhang Q., Abdul Rehman R.M. (2018). Alternative splicing of *OsLG3b* controls grain length and yield in japonica rice. Plant Biotechnol. J..

[12] Song X.J., Huang W., Shi M., Zhu M.Z., Lin H.X. (2007). A QTL for rice grain width and weight encodes a previously unknown RING-type E3 ubiquitin ligase. Nat. Genet..

[13] Song X.J., Kuroha T., Ayano M., Furuta T., Nagai K., Komeda N., Segami S., Miura K., Ogawa D., Kamura T. (2015). Rare allele of a previously unidentified histone H4 acetyltransferase enhances grain weight, yield, and plant biomass in rice. Proc. Natl. Acad. Sci. USA.

[14] Shomura A., Izawa T., Ebana K., Ebitani T., Kanegae H., Konishi S., Yano M. (2008). Deletion in a gene associated with grain size increased yields during rice domestication. Nat. Genet..

[15] Li Y., Fan C., Xing Y., Jiang Y., Luo L., Sun L., Shao D., Xu C., Li X., Xiao J. (2011). Natural variation in *GS5* plays an important role in regulating grain size and yield in rice. Nat. Genet..

[16] Wang S., Wu K., Yuan Q., Liu X., Liu Z., Lin X., Zeng R., Zhu H., Dong G., Qian Q. (2012). Control of grain size, shape and quality by *OsSPL16* in rice. Nat. Genet..

[17] Liu J., Chen J., Zheng X., Wu F., Lin Q., Heng Y., Tian P., Cheng Z., Yu X., Zhou K. (2017). *GW5* acts in the brassinosteroid signalling pathway to regulate grain width and weight in rice. Nat. Plants.

[18] Ruan B., Shang L., Zhang B., Hu J., Wang Y., Lin H., Zhang A., Liu C., Peng Y., Zhu L. (2020). Natural variation in the promoter of *TGW2* determines grain width and weight in rice. New Phytol..

[19] Che R., Tong H., Shi B., Liu Y., Fang S., Liu D., Xiao Y., Hu B., Liu L., Wang H. (2015). Control of grain size and rice yield by *GL2*-mediated brassinosteroid responses. Nat. Plants.

[20] Hu J., Wang Y., Fang Y., Zeng L., Xu J., Yu H., Shi Z., Pan J., Zhang D., Kang S. (2015). A rare allele of *GS2* enhances grain size and grain yield in rice. Mol. Plant.

[21] Si L., Chen J., Huang X., Gong H., Luo J., Hou Q., Zhou T., Lu T., Zhu J., Shangguan Y. (2016). *OsSPL13* controls grain size in cultivated rice. Nat. Genet..

[22] Wang A., Hou Q., Si L., Huang X., Luo J., Lu D., Zhu J., Shangguan Y., Miao J., Xie Y. (2019). The PLATZ transcription factor *GL6* affects grain length and number in rice. Plant Physiol..

[23] Dong N.Q., Sun Y., Guo T., Shi C.L., Zhang Y.M., Kan Y., Xiang Y.H., Zhang H., Yang Y.B., Li Y.C. (2020). UDP-glucosyltransferase regulates grain size and abiotic stress tolerance associated with metabolic flux redirection in rice. Nat. Comm..

[24] Wang S., Li S., Liu Q., Wu K., Zhang J., Wang S., Wang Y., Chen X., Zhang Y., Gao C. (2015). The *OsSPL16-GW7* regulatory module determines grain shape and simultaneously improves rice yield and grain quality. Nat. Genet..

[25] Wang Y., Xiong G., Hu J., Jiang L., Yu H., Xu J., Fang Y., Zeng L., Xu E., Xu J. (2015). Copy number variation at the *GL7* locus contributes to grain size diversity in rice. Nat. Genet..

[26] Zhao D.S., Li Q.F., Zhang C.Q., Zhang C., Yang Q.Q., Pan L.X., Ren X.Y., Lu J., Gu M.H., Liu Q.Q. (2018). *GS9* acts as a transcriptional activator to regulate rice grain shape and appearance quality. Nat. Comm..

[27] Yan S., Zou G., Li S., Wang H., Liu H., Zhai G., Guo P., Song H., Yan C., Tao Y. (2011). Seed size is determined by the combinations of the genes controlling different seed characteristics in rice. Theor. Appl. Genet..

[28] Rath D., Amlinger L., Rath A., Lundgren M. (2015). The CRISPR-Cas immune system: Biology, mechanisms and applications. Biochimie.

[29] Belhaj K., Chaparro-Garcia A., Kamoun S., Patron N.J., Nekrasov V. (2015). Editing plant genomes with CRISPR/Cas9. Curr. Opin. Biotechnol..

[30] Osakabe Y., Osakabe K. (2015). Genome editing with engineered nucleases in plants. Plant Cell Physiol..

[31] Komor A.C., Badran A.H., Liu D.R. (2017). CRISPR-based technologies for the manipulation of eukaryotic genomes. Cell.

[32] Marraffini L.A., Sontheimer E.J. (2010). CRISPR interference: RNA-directed adaptive immunity in bacteria and archaea. Nat. Rev. Genet..

[33] Sander J.D., Joung J.K. (2014). CRISPR-Cas systems for editing, regulating and targeting genomes. Nat. Biotechnol..

[34] Jinek M., Chylinski K., Fonfara I., Hauer M., Doudna J.A., Charpentier E. (2012). A Programmable Dual-RNA-Guided DNA Endonuclease in Adaptive Bacterial Immunity. Science.

[35] Feng Z., Mao Y., Xu N., Zhang B., Wei P., Yang D.L., Wang Z., Zhang Z., Zheng R., Yang L. (2014). Multigeneration analysis reveals the inheritance, specificity, and patterns of CRISPR/Cas-induced gene modifications in Arabidopsis. Proc. Natl. Acad. Sci. USA.

[36] Shan Q., Wang Y., Li J., Zhang Y., Chen K., Liang Z., Zhang K., Liu J., Xi J.J., Qiu J.L. (2013). Targeted genome modification of crop plants using a CRISPR-Cas system. Nat. Biotechnol..

[37] Li J.F., Norville J.E., Aach J., McCormack M., Zhang D., Bush J., Church G.M., Sheen J. (2013). Multiplex and homologous recombination–mediated genome editing in Arabidopsis and Nicotiana benthamiana using guide RNA and Cas9. Nat. Biotechnol..

[38] Nekrasov V., Staskawicz B., Weigel D., Jones J.D., Kamoun S. (2013). Targeted mutagenesis in the model plant Nicotiana benthamiana using Cas9 RNA-guided endonuclease. Nat. Biotechnol..

[39] Mao Y., Zhang H., Xu N., Zhang B., Gou F., Zhu J.-K. (2013). Application of the CRISPRG Cas system for efficient genome engineering in plants. Mol. Plant.

[40] Liang Z., Zhang K., Chen K., Gao C. (2014). Targeted mutagenesis in *Zea mays* using TALENs and the CRISPR/Cas system. J. Genet. Genom..

[41] Xing H.L., Dong L., Wang Z.P., Zhang H.Y., Han C.Y., Liu B., Wang X.C., Chen Q.J. (2014). A CRISPR/Cas9 toolkit for multiplex genome editing in plants. BMC Plant Biol..

[42] Jacobs T.B., LaFayette P.R., Schmitz R.J., Parrott W.A. (2015). Targeted genome modifications in soybean with CRISPR/Cas9. BMC Biotechnol..

[43] Liao S., Qin X., Luo L., Han Y., Wang X., Usman B., Nawaz G., Zhao N., Liu Y., Li R. (2019). CRISPR/Cas9-Induced Mutagenesis of *Semi-Rolled Leaf1, 2* Confers Curled Leaf Phenotype and Drought Tolerance by Influencing Protein Expression Patterns and ROS Scavenging in Rice (*Oryza sativa* L.). Agronomy.

[44] Han Y., Teng K., Nawaz G., Feng X., Usman B., Wang X., Luo L., Zhao N., Liu Y., Li R. (2019). Generation of semi-dwarf rice (*Oryza sativa* L.) lines by CRISPR/Cas9-directed mutagenesis of *OsGA20ox2* and proteomic analysis of unveiled changes caused by mutations. 3 Biotech.

[45] Nawaz G., Usman B., Peng H., Zhao N., Yuan R., Liu Y., Li R. (2020). Knockout of *Pi21* by CRISPR/Cas9 and iTRAQ-Based Proteomic Analysis of Mutants Revealed New Insights into *M. oryzae* Resistance in Elite Rice Line. Genes.

[46] Nawaz G., Han Y., Usman B., Liu F., Qin B., Li R. (2019). Knockout of *OsPRP1*, a gene encoding proline-rich protein, confers enhanced cold sensitivity in rice (*Oryza sativa* L.) at the seedling stage. 3 Biotech.

[47] Han Y., Luo D., Usman B., Nawaz G., Zhao N., Liu F., Li R. (2018). Development of high yielding glutinous cytoplasmic male sterile rice (*Oryza sativa* L.) lines through CRISPR/Cas9 based mutagenesis of *Wx* and *TGW6* and proteomic analysis of anther. Agronomy.

[48] Usman B., Nawaz G., Zhao N., Liu Y., Li R. (2020). Generation of High Yielding and Fragrant Rice (*Oryza sativa* L.) Lines by CRISPR/Cas9 Targeted Mutagenesis of Three Homoeologs of Cytochrome P450 Gene Family and *OsBADH2* and Transcriptome and Proteome Profiling of Revealed Changes Triggered by Mutations. Plants.

[49] Nawaz G., Usman B., Zhao N., Han Y., Li Z., Wang X., Liu Y., Li R. (2020). CRISPR/Cas9 Directed Mutagenesis of *OsGA20ox2* in High Yielding Basmati Rice (*Oryza sativa* L.) Line and Comparative Proteome Profiling of Unveiled Changes Triggered by Mutations. Int. J. Mol. Sci..

[50] Usman B., Nawaz G., Zhao N., Liao S., Liu Y., Li R. (2020). Precise Editing of the *OsPYL9* Gene by RNA-Guided Cas9 Nuclease Confers Enhanced Drought Tolerance and Grain Yield in Rice (*Oryza sativa* L.) by Regulating Circadian Rhythm and Abiotic Stress Responsive Proteins. Int. J. Mol. Sci..

[51] Weng J., Gu S., Wan X., Gao H., Guo T., Su N., Lei C., Zhang X., Cheng Z., Guo X. (2008). Isolation and initial characterization of *GW5*, a major QTL associated with rice grain width and weight. Cell Res..

[52] Zhou J., Xin X., He Y., Chen H., Li Q., Tang X., Zhong Z., Deng K., Zheng X., Akher S.A. (2019). Multiplex QTL editing of grain-related genes improves yield in elite rice varieties. Plant Cell Rep..

[53] Zhang Y., Li D., Zhang D., Zhao X., Cao X., Dong L., Liu J., Chen K., Zhang H., Gao C. (2018). Analysis of the functions of *TaGW2* homoeologs in wheat grain weight and protein content traits. Plant J..

[54] Zeng Y., Wen J., Zhao W., Wang Q., Huang W. (2020). Rational Improvement of Rice Yield and Cold Tolerance by Editing the Three Genes *OsPIN5b*, *GS3*, and *OsMYB30* With the CRISPR–Cas9 System. Front. Plant Sci..

[55] Zhang H., Zhang J., Liang Z., Botella J.R., Zhu J.K. (2017). Genome editing—principles and applications for functional genomics research and crop improvement. Crit. Rev. Plant Sci..

[56] Li M., Li X., Zhou Z., Wu P., Fang M., Pan X., Lin Q., Luo W., Wu G., Li H. (2016). Reassessment of the four yield-related genes Gn1a, DEP1, GS3, and IPA1 in rice using a CRISPR/Cas9 system. Front. Plant Sci..

[57] Xu R., Yang Y., Qin R., Li H., Qiu C., Li L., Wei P., Yang J. (2016). Rapid improvement of grain weight via highly efficient CRISPR/Cas9-mediated multiplex genome editing in rice. J. Genet. Genom..

[58] Yang Y., Chen X., Xu B., Li Y., Ma Y., Wang G. (2015). Phenotype and transcriptome analysis reveals chloroplast development and pigment biosynthesis together influenced the leaf color formation in mutants of Anthurium andraeanum ‘Sonate’. Front. Plant Sci..

[59] Hellinger R., Koehbach J., Soltis D.E., Carpenter E.J., Wong G.K.S., Gruber C.W. (2015). Peptidomics of circular cysteine-rich plant peptides: Analysis of the diversity of cyclotides from viola tricolor by transcriptome and proteome mining. J. Proteome Res..

[60] Kamal A.H.M., Cho K., Choi J.S., Bae K.H., Komatsu S., Uozumi N., Woo S.H. (2013). The wheat chloroplastic proteome. J. Protome.

[61] Bibikova M., Golic M., Golic K.G., Carroll D. (2002). Targeted chromosomal cleavage and mutagenesis in Drosophila using zinc-finger nucleases. Genetics.

[62] Bibikova M., Beumer K., Trautman J.K., Carroll D. (2003). Enhancing gene targeting with designed zinc finger nucleases. Science.

[63] Dreier B., Fuller R.P., Segal D.J., Lund C.V., Blancafort P., Huber A., Koksch B., Barbas C.F. (2005). Development of zinc finger domains for recognition of the 5′-CNN-3′ family DNA sequences and their use in the construction of artificial transcription factors. J. Biol. Chem..

[64] Hockemeyer D., Wang H., Kiani S., Lai C.S., Gao Q., Cassady J.P., Cost G.J., Zhang L., Santiago Y., Miller J.C. (2011). Genetic engineering of human pluripotent cells using TALE nucleases. Nat. Biotechnol..

[65] Tesson L., Usal C., Ménoret S., Leung E., Niles B.J., Remy S., Santiago Y., Vincent A.I., Meng X., Zhang L. (2011). Knockout rats generated by embryo microinjection of TALENs. Nat. Biotechnol..

[66] Huang P., Xiao A., Zhou M., Zhu Z., Lin S., Zhang B. (2011). Heritable gene targeting in zebrafish using customized TALENs. Nat. Biotechnol..

[67] Zhang H., Zhang J., Wei P., Zhang B., Gou F., Feng Z., Mao Y., Yang L., Zhang H., Xu N. (2014). The CRISPR/C as9 system produces specific and homozygous targeted gene editing in rice in one generation. Plant Biotechnol. J..

[68] Ambasht P., Kayastha A.M. (2002). Plant pyruvate kinase. Biol. Planta..

[69] Valentini G., Chiarelli L., Fortin R., Speranza M.L., Galizzi A., Mattevi A. (2000). The allosteric regulation of pyruvate kinase A site-directed mutagenesis study. J. Biol. Chem..

[70] Zhang B., Liu J.Y. (2016). Cotton cytosolic pyruvate kinase *GhPK6* participates in fast fiber elongation regulation in a ROS-mediated manner. Planta.

[71] Zhang Y., Xiao W., Luo L., Pang J., Rong W., He C. (2012). Downregulation of *OsPK1*, a cytosolic pyruvate kinase, by T-DNA insertion causes dwarfism and panicle enclosure in rice. Planta.

[72] Cai Y., Li S., Jiao G., Sheng Z., Wu Y., Shao G., Xie L., Peng C., Xu J., Tang S. (2018). *OsPK 2* encodes a plastidic pyruvate kinase involved in rice endosperm starch synthesis, compound granule formation and grain filling. Plant Biotechnol. J..

[73] Herrera I., De La Paz Sánchez1 M., Molina J., Plasencia J., Vázquez-Ramos J.M. (2000). Proliferating cell nuclear antigen expression in maize seed development and germination: Regulation by phytohormones and its association with putative cell cycle proteins. Physiol. Planta..

[74] Huang X., Qian Q., Liu Z., Sun H., He S., Luo D., Xia G., Chu C., Li J., Fu X. (2009). Natural variation at the *DEP1* locus enhances grain yield in rice. Nat. Genet..

[75] Duan P., Rao Y., Zeng D., Yang Y., Xu R., Zhang B., Dong G., Qian Q., Li Y. (2014). *SMALL GRAIN 1*, which encodes a mitogen-activated protein kinase kinase 4, influences grain size in rice. Plant J..

[76] Liu S., Hua L., Dong S., Chen H., Zhu X., Jiang J.E., Zhang F., Li Y., Fang X., Chen F. (2015). Os MAPK 6, a mitogen-activated protein kinase, influences rice grain size and biomass production. Plant J..

[77] Liu L., Tong H., Xiao Y., Che R., Xu F., Hu B., Liang C., Chu J., Li J., Chu C. (2015). Activation of Big Grain1 significantly improves grain size by regulating auxin transport in rice. PNAS.

[78] Li S., Liu W., Zhang X., Liu Y., Li N., Li Y. (2012). Roles of the Arabidopsis G protein γ subunit AGG3 and its rice homologs GS3 and DEP1 in seed and organ size control. Plant Signal. Behav..

[79] Xie X., Ma X., Zhu Q., Zeng D., Li G., Liu Y.G. (2017). CRISPR-GE: A convenient software toolkit for CRISPR-based genome editing. Mol. Plant.

[80] Ma X., Zhang Q., Zhu Q., Liu W., Chen Y., Qiu R., Wang B., Yang Z., Li H., Lin Y. (2015). A robust CRISPR/Cas9 system for convenient, high-efficiency multiplex genome editing in monocot and dicot plants. Mol. Plant.

[81] Hiei Y., Ohta S., Komari T., Kumashiro T. (1994). Efficient transformation of rice (*Oryza sativa* L.) mediated by Agrobacterium and sequence analysis of the boundaries of the T-DNA. Plant J..

[82] Liu W., Xie X., Ma X., Li J., Chen J., Liu Y.G. (2015). DSDecode: A web-based tool for decoding of sequencing chromatograms for genotyping of targeted mutations. Mol. Plant.

[83] Wang Z.Q., Xu X.Y., Gong Q.Q., Xie C., Fan W., Yang J.L., Lin Q.S., Zheng S.J. (2014). Root proteome of rice studied by iTRAQ provides integrated insight into aluminum stress tolerance mechanisms in plants. J. Proteome.

[84] Livak K.J., Schmittgen T.D. (2001). Analysis of relative gene expression data using real-time quantitative PCR and the 2−ΔΔCT method. Methods.

