# Supplementary material for: Programmed Editing of Rice (Oryza sativa L.) OsSPL16 Gene Using CRISPR/Cas9 Improves Grain Yield by Modulating the Expression of Pyruvate Enzymes and Cell Cycle Proteins
Source: Int J Mol Sci. 2020 Dec 29;22(1):249. doi: 10.3390/ijms22010249 (PMC7795130; doi:10.3390/ijms22010249)
Supplement: Supplementary file 1 [file ijms-22-00249-s001.zip › Supplementary file 1.docx]

**Table S1.** List of primers used for the construction of vector and genotyping of mutant plants.

| **Primer name** | **Primer Sequence (5'-3')** |
| --- | --- |
| qGW8F/R | F: ACGGAATCGAGAGTGAGGAGGC |
|  | R: TTTGGCGGGAAGGAAGGAGACA |
| gRT1: | CCGACGAGCTGGAGAACAGgttttagagctagaaat |
| OsU6aT1: | CTGTTCTCCAGCTCGTCGGCggcagccaagccagca |
| gRT2: | GTAGTCGCGGCACTTGCTCgttttagagctagaaat |
| OsU6aT2: | GAGCAAGTGCCGCGACTACCaacacaagcggcagc |
| U-F | CTCCGTTTTACCTGTGGAATCG |
| gR-R | CGGAGGAAAATTCCATCCAC |
| Pps-R | TTCAGA**ggtctc**T**ACCG**ACTAGTATGGAATCGGCAGCAAAGG |
| Pgs-2 | AGCGTG**ggtctc**G**tcag**ggTCCATCCACTCCAAGCTC |
| Pps-2 | TTCAGA**ggtctc**T**ctga**cacTGGAATCGGCAGCAAAGG |
| Pgs-L | AGCGTGGGTCTCGTCTTCACTCCATCCACTCCAAGCTC |
| PB-R | GCGCGCGGTCTCTACCGACGCGTATCC |
| PB-L | GCGCGCgGTCTCGCTCGACTAGTATGG |
| HPT | F: GTGCTTGACATTGGGGAGTT  R: ATTTGTGTACGCCCGACAGT |
| Cas9-F | CTGACGCTAACCTCGACAAG |
| Cas9-R | CCGATCTAGTAACATAGATGACACC |
| SP-L1 | GCGGTGTCATCTATGTTACTAG |
| SP-R | GCCTATACCAAGTTATTGCA |

**Table S2.** Mutations types in T_0_ events achieved by transformation with two CRISPR/Cas9 constructs.

| **Sr. No.** | **Genotype** | **Target 1** | **Target 2** |
| --- | --- | --- | --- |
| 1 | GXU52-1 | -3/- | -2/- |
| 2 | GXU52-2 | -2/-2 | WT |
| 3 | GXU52-3 | -4/- | -3/- |
| 4 | GXU52-4 | WT | WT |
| 5 | GXU52-5 | -2/- | -1/-1 |
| 6 | GXU52-6 | -6/-6 | -7/-7 |
| 7 | GXU52-7 | WT | WT |
| 8 | GXU52-8 | -9/-9 | -3/-3 |
| 9 | GXU52-9 | -10/-2 | -3/- |
| 10 | GXU52-10 | WT | WT |
| 11 | GXU52-11 | -1, +1/- | -2/-1 |
| 12 | GXU52-12 | -3/-2 | -2/- |

^_^: deletion, +: insertion. The numbers in front of the dashes indicate the number of nucleotides affected. Corresponding mutations in both alleles are distinguished by ‘/’.

**Table S3.** Primers designed for off-target sites evaluation.

| **Primer name** | **Primer Sequence (5'-3')** |
| --- | --- |
| POT1 | F: CCATGCCCTTCATCATAGGT  R: GAACACCTGCGTCAGGTACA |
| POT2 | F: GAACGGCGAGAGGCAGAC  R: GTGGGGAGGAGCCATGAG |
| POT3 | F: GGTCGGTGGTGTACGTGAG  R: GACTCGTGCTTCAGGACCTC |
| POT4 | F: AAGGCAGCCAACAAAATCAG  R: CTCTTCCCTCTCCTCGCTCT |
| POT5 | F: ACGACGAGGAGGAGGACAG  R: ACTCGGGGGTCTGTGAAAAT |
| POT6 | F: TGCCAAATTTTACCCGATTT  R: AAGCACCGCGACTACCAC |
| POT7 | F: CCATAGGCTGCTCAGGTAGC  R: CAGATGACAACGGCTCAGAA |
| POT8 | F: AGTTGTTCGGGTCGTTGTTC  R: AAGTACATGACCAGCCTGACG |
| POT9 | F: TTGCAGTAGTCGTCGGTGAG  R: CTGCAGGTGGCCATGAAG |
| POT10 | F: GGTTGCATGTGACGTTGAAG  R: AAATGGCGGTGAAGAAACAC |

**Table S4.** Mutations detection on five most likely putative off-target sites.

| **Target** | **NOPO** | **GL** | **Locus** | **Sequence** | **MMB** | **NPS** | **NPOM** | **OTS** | **Region** |
| --- | --- | --- | --- | --- | --- | --- | --- | --- | --- |
| T1 | OT1 | Chr3: 27418594-27418616 | LOC_Os03g48180 | G**T**CGAC**A**AGCTGGAGAAC**G**G **CGG** | 3 | 18 | 0 | 0.341 | CDS |
|  | OT2 | Chr2: 28246825-28246847 | LOC_Os02g46340 | GCCGACG**CCA**TGG**T**GAACAG **CGG** | 4 | 18 | 0 | 0.107 | CDS |
|  | OT3 | Chr6: 10480232-10480254 | LOC_Os06g18010 | GCCG**C**CG**G**GCTGGAGA**C**CAG **CGG** | 3 | 18 | 0 | 0.065 | CDS |
|  | OT4 | Chr2: 15142820-15142842 | LOC_Os02g25840 | GC**G**GACGAG**GC**GGAGAAC**G**G **CGG** | 4 | 18 | 0 | 0.049 | CDS |
|  | OT5 | Chr6: 25419211-25419233 | LOC_Os06g42320 | G**A**CGACGAG**GA**GGAG**G**ACAG **CGG** | 4 | 18 | 0 | 0.041 | CDS |
| T2 | OT6 | Chr9: 19643831-19643853 | LOC_Os09g32944 | GGTAGTCGCGG**TG**CTTGCTC **AGG** | 2 | 18 | 0 | 0.351 | CDS |
|  | OT7 | Chr3: 26582051-26582073 | LOC_Os03g47016 | GGTAG**C**CGC**T**G**T**ACTTG**T**TC **AGG** | 4 | 18 | 0 | 0.256 | CDS |
|  | OT8 | Chr10: 96035-96057 | LOC_Os10g01100 | **C**GT**T**G**G**C**C**CGGCACTTGCTC **TGG** | 4 | 18 | 0 | 0.160 | CDS |
|  | OT9 | Chr5: 19446175-19446197 | LOC_Os05g33150 | GG**G**AG**A**CGCGGCACT**G**GCTC **TGG** | 3 | 18 | 0 | 0.150 | CDS |
|  | OT10 | Chr11: 28357189-28357211 | LOC_Os11g47140 | **C**G**AC**GTCGC**C**GCACTTGCTC **GGG** | 4 | 18 | 0 | 0.072 | CDS |

Note: The protospacer adjacent motif (PAM) (NGG) is shown in green background. NOPO; name of putative off-target, GL; genomic location, MMB; mis-matching bases, NPS; number of plants screened, NPOM; number of plants with off-target mutations, OTS; off-target score. T1 and T2 represents target1 and target2.

**Table S5.** Segregation pattern of homozygous, mono-allelic heterozygous, and bi-allelic heterozygous mutations in T_1_ generation.

| **Targets** | **T_0_** | | | **T_1_** | | | | |
| --- | --- | --- | --- | --- | --- | --- | --- | --- |
|  | **Mutants** | **Zygosity** | **InDels** | **PT** | **WT** | **Bi** | **Homo** | **Hetero** |
| T1 | GXU52-6 | Hom | 6d/6d | 20 | 0 | 0 | 20(6d/6d) | 0 |
| T2 | GXU52-6 | Hom | 7d/7d | 20 | 0 | 0 | 20(7d/7d) | 0 |
| T1 | GXU52-3 | Mono Het | 4d/- | 42 | 11 | 0 | 21(4d/4d) | 10(WT/WT) |
| T2 | GXU52-3 | Mono Het | 3d/- | 42 | 13 | 0 | 20(3d/3d) | 9(WT/WT) |
| T1 | GXU52-9 | Bi Het | 10d/2d | 42 | 0 | 21(10d/2d) | 11(10d), 10(2d) | 0 |
| T2 | GXU52-11 | Bi Het | 2d/1d | 42 | 0 | 21(2d/1d) | 10(2d), 11(1d) | 0 |

PT; number of plants tested, WT; wild type, Bi Het; bi-allelic heterozygous, Homo; homozygous, Mono Het; mono-allelic heterozygous, Chi; chimeric. d: deletion, i: insertion and WT: wild type. The numbers in front of the letters indicate the number of nucleotides affected. Corresponding mutations in two alleles are distinguished by ‘/’.

**Table S6.** Selected target positions with their GC content and potential off-target score.

| **Target** | **Target Sequence (5’-3’)** | **Position** | **Strand** | **GC %** | **Region** | **Off-target Score** | **Pairing with sgRNA (>=8 nt)** |
| --- | --- | --- | --- | --- | --- | --- | --- |
| T1 | GCCGACGAGCTGGAGAACAG**CGG** | 102-111 | + | 65.0 | CDS | 0.341 | None |
| T2 | GGTAGTCGCGGCACTTGCTC**AGG** | 456-437 | - | 65.0 | CDS | 0.351 | None |

T1 and T2; represent the Target1 and Target2. Green highlighted are PAM regions.

**Table S7.** Primers designed for RT-qPCR analysis.

| **Gene ID** | **Forward Primer (3’-5’)** | **Reverse Primer (5’-3’)** |
| --- | --- | --- |
| *OsSPL16* | AGGAGTTTGATGAGGCCAAG | GCGTGTAGTATGGGCTCTCC |
| *OsBADH2* | AGAGACGCTTGATTGTGGGA | ACAGTGGATAACTGGCCACA |
| *Os02g0589400* | TCGACGCTTTCATTCAGCAG | TAGCAAACTTGGCAAGCACT |
| *PDRP1* | TCCACGAAGTTCTCCTAGCC | TGTGTGCACGGTTAAGGTTC |
| *CSP2* | CCCTCCCTCACTGAATCACC | TCAAACCGACCCAACGAAAC |
| *OsPPDKB* | GTTCATGGCACCAAACACCT | TTTGCTCAGCGATTGCGTTA |
| *Os02g0739600* | GCCTTCGCGCAGAAGTATAG | GGCACAGGAACTCACAATGG |
| *Os06g0105400* | AAGCGACAGTGGAGATGGAA | TCAGCACAGAACATGGGCTA |
| *Os12g0182200* | CCTTCACAACCGATGCTCTG | ACCATCCCACCACTGCATAA |
| *Os02g0701600* | CCATAGGAACCTCGAGCCAT | AAGAGACAGTGACTCGGCAA |
| *Os03g0784800* | ACATTGCCAGTTACCATCGC | CCAACGCCTTGTGACATGAT |
| *OsActin1* | TTCCTCATGCCATCCTGCGTCTG | GTCCCTTACAATTTCCCGTTCAGC |


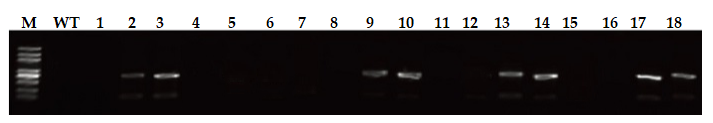


**Figure S1.** Screening of Transgene (T-DNA) free plants using Cas9 specific primers. Two randomly selected plants from each mutant line were evaluated. WT represents a wild type, whereas, 1-18 numbers represent mutant line from GXU52-1-1 to GXU52-9-2. The mutant lines that failed to amplify to the target sequence were termed as T-DNA-free.

**
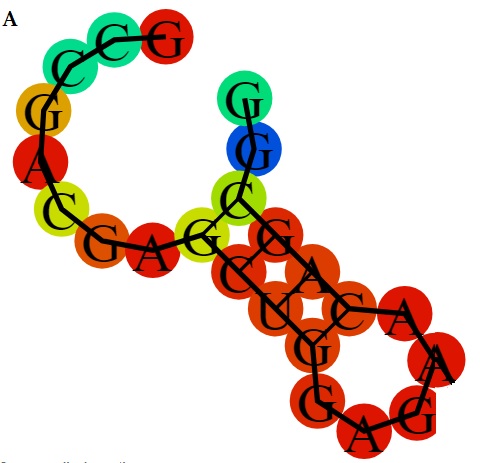

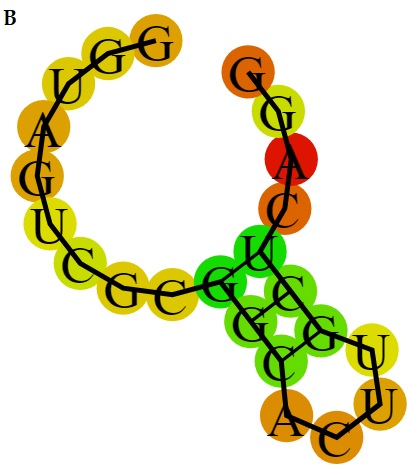
**

**Figure S2.** Schematic representation of secondary structures of **(A)** sgRNA1; and **(B)** sgRNA2; used in the experiment.
